# Abuse Potential of Cathinones in Humans: A Systematic Review

**DOI:** 10.3390/jcm11041004

**Published:** 2022-02-15

**Authors:** Lourdes Poyatos, Adrián Torres, Esther Papaseit, Clara Pérez-Mañá, Olga Hladun, Melani Núñez-Montero, Georgina de la Rosa, Marta Torrens, Daniel Fuster, Robert Muga, Magí Farré

**Affiliations:** 1 Department of Clinical Pharmacology, Hospital Universitari Germans Trias i Pujol and Institut de Recerca Germans Trias i Pujol (HUGTiP-IGTP), 08916 Badalona, Spain; lpoyatos@igtp.cat (L.P.); cperezm.mn.ics@gencat.cat (C.P.-M.); ohladun.germanstrias@gencat.cat (O.H.); mnunezmon.germanstrias@gencat.cat (M.N.-M.); grosalo.germanstrias@gencat.cat (G.d.l.R.); 2 Department of Pharmacology, Therapeutics and Toxicology, Universitat Autònoma de Barcelona (UAB), 08913 Cerdanyola del Vallés, Spain; adrian.torresv@e-campus.uab.cat; 3 Institut de Neuropsiquiatria i Adiccions (INAD), Parc de Salut Mar, 08003 Barcelona, Spain; mtorrens@parcdesalutmar.cat; 4 Department of Psychiatry and Legal Medicine, Universitat Autònoma de Barcelona (UAB), 08913 Cerdanyola del Vallés, Spain; 5 Department of Internal Medicine, Hospital Universitari Germans Trias i Pujol and Institut de Recerca Germans Trias i Pujol (HUGTiP-IGTP), 08916 Badalona, Spain; dfuster.germanstrias@gencat.cat (D.F.); rmuga.germanstrias@gencat.cat (R.M.); 6 Department of Medicine, Universitat Autònoma de Barcelona (UAB), 08913 Cerdanyola del Vallés, Spain

**Keywords:** cathinone, mephedrone (4-methylmethcathinone), methylone (3,4-methylenedioxymethcathinone), diethylpropion, abuse potential, new psychoactive substance, pharmacological effects, pharmacokinetics

## Abstract

*Introduction and objective*: Assessing the abuse potential of new substances with central nervous system activity is essential for preventing possible risks of misuse and addiction. The same methodology is recommended for the evaluation of the abuse potential of recreational drugs. This systematic review aims to assess the pharmacological effects related to the abuse potential and pharmacokinetics of cathinones, which are evaluated in both experimental and prospective observational studies in humans. *Materials and Methods*: A systematic search of the published literature was conducted to retrieve studies that had administered cathinone, mephedrone, methylone, and diethylpropion to evaluate their acute pharmacological effects related to abuse potential. *Results*: The search yielded 583 results, 18 of which were included to assess the abuse potential of cathinone (*n* = 5), mephedrone (*n* = 7), methylone (*n* = 1), and diethylpropion (*n* = 5). All four substances induce stimulant and euphorigenic effects that resemble those of amphetamines and MDMA, and their different intensities may be associated with varying levels of abuse potential. *Conclusions*: Cathinone, mephedrone, methylone, and diethylpropion induce a range of desirable and reinforcing effects that may, to some extent, result in abuse potential. Further investigation is needed to minimize and prevent their impact on society and public health.

## 1. Introduction

Evaluating the abuse potential of new substances, particularly that of those that are able to affect the central nervous system, is necessary to determine future prescription conditions and the possible risk of misuse and addiction. In fact, the Prescriber Information (PI for US-FDA) and the Summary of Product Characteristics (SPC in Europe-EMA) include a specific section on this topic.

Research assessing abuse potential is performed in accordance with the methodology recommended by the Food and Drug Administration (FDA) in its industry guidelines entitled “Assessment of abuse potential of drugs”, published in 2017 [1]. Such studies in humans are considered Phase I clinical trials and include healthy recreational and regular substance users. In a similar manner to other Phase I trials, volunteers gain no therapeutic benefit from their participation. These studies are therefore typically carried out in specialized units of human/clinical pharmacology. The methodology that is implemented for abuse potential assessment has been well established. It includes clinical trials that compare the subjective effects (such as evaluation of euphoria, liking, and well-being) that are induced by a known substance (positive control) compared to the new substance being evaluated and a placebo. Most abusable substances are generally introduced into the illegal market without specific studies and/or information. As a result, the methodology that has been described for medicines is used to evaluate the relative abuse potential of the new psychoactive substances compared to well-known drugs in order to recognize their abuse liability/potential.

Abuse or addiction to a substance/medicine is an adverse drug reaction which, according to European and North American legislation, is defined as “A response to a medicinal product which is noxious and unintended” [2]. In a similar manner to other adverse drug reactions, the misuse, abuse, and addiction of a substance can be prevented by previous information and prescription limitations. Thus, it reinforces the need for experimental studies of abuse potential in animals and humans.

As previously mentioned, the majority of new psychoactive substances are introduced into the illegal market and are consumed by an ever-increasing number of individuals. In most cases, these compounds become illegal due their deleterious health effects, including emergency cases and lethality. As an example, the cathinone derivative mephedrone (4-methylmethcathinone) arrived on the market in 2008. It was made illegal in 2010 in Europe and in 2011 in the United States after being used by millions around the world; the only data on its effects, however, were obtained from surveys and intoxication cases series [3]. The first Phase I clinical trial evaluating mephedrone abuse potential and pharmacokinetics in humans was published in 2016, five years after it was outlawed. This study was essential for understanding its patterns of use and abuse potential compared to MDMA.

The aim of this study is to evaluate the pharmacological effects related to the abuse potential and pharmacokinetic properties of cathinone and synthetic cathinones by reviewing their origins, chemistry, pharmacodynamics, and pharmacokinetics using a standardized systematic review procedure.

### 1.1. Cathinones from Plant to Synthetics

Khat (*Catha edulis* Forsk., Celastraceae), also known as qat, is a flowering plant that is native to Ethiopia, although it has also been widely cultivated and consumed in parts of East Africa and the Arabian Peninsula [4,5,6,7]. The fresh khat leaves are usually chewed to form a bolus (quid) to obtain stimulant and euphoric effects. In these countries, khat chewing represents a deep-rooted tradition that is undertaken to avoid hunger and fatigue as well as to ease social interaction [4,8]. This habit is a typical activity that is performed during social khat sessions, in which each participant commonly chews about 100–200 g of leaves [9]. Khat can also be ingested by making a drink from dried leaves, or more rarely, by smoking dried ones [10]. Beyond its traditional use, khat chewing has become popular among university students seeking to enhance their academic performance and to reduce fatigue [11,12]. The legal status of khat varies by region depending on its traditional use in some cultures, although it can be illegal under more general laws [6].

Khat use has spread to western countries. It can be found in major cities in the United States, Europe, and Australia, especially among immigrant communities that maintain this habit [6,13]. According to some authorities, the estimated number of individuals chewing fresh khat leaves on a regular basis ranges from 10 to 20 million, with almost half of whom doing so on a daily basis [5,6,14]. This finding indicates that it is possibly the most widely used psychoactive herb in the world [7].

In the previous decade, new psychoactive substances (NPS), also called legal highs, have become increasingly popular; according to United Nations Office on Drug and Crime (UNODC), the center of the global NPS market emerged in North America and Western and Central Europe, but it has moved to Asia, Africa, and Latin American in recent years [15]. These include non-illegal recreational alternatives that exploit the inadequacies of existing controlled substance legislation [16,17]. According to the European Drug report, synthetic cannabinoids and synthetic cathinones are the most seized NPS. In 2019, they represented 60% of seizures, although the prevalence of use of each individual substance is a minority [18]. Some data clarifying the trends and prevalence of use of synthetic cathinones are provided by wastewater monitoring programs. For instance, methylone and mephedrone have been detected in variable low levels in several analyzed areas of Australia. Mephedrone and methylone consumption were found to be higher during weekends, a pattern that is consistent with the patterns for other illicit drugs such as cocaine and MDMA (3,4-methylenedioxymethamphetamine) [19].

### 1.2. Chemistry

**Figure 1:**
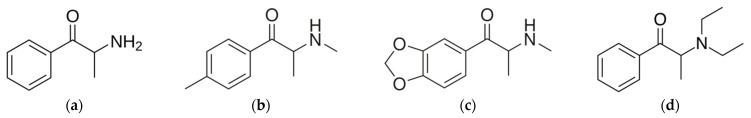
Molecular structures of cathinone (**a**), mephedrone (**b**), methylone (**c**), and diethylpropion (**d**).

The main psychoactive alkaloid in khat is the phenylpropylamine derivative cathinone (S-(-)-α-aminopropiophenone) (Figure 1), which comprises khat together with the less psychoactive phenylpropanolamine diastereomers cathine (S,S-(+)-nor-pseudoephedrine) and R,S-(-)-norephedrine [5,20].

In recent years, a number of substituted cathinones have been introduced onto the drug black market, with some of the most relevant being mephedrone, methylone, ethylone, euthylone, and methylenedioxypyrovalerone (MDPV). However, not all synthetic cathinones are classified as abuse substances based on their psychostimulant effects and abuse potential. For instance, diethylpropion (or amfepramone) was marketed as an appetite suppressant for the short-term management of obesity (schedule IV controlled substance) for years, and bupropion is prescribed as an atypical antidepressant and treatment for smoking cessation (uncontrolled substance) [21].

### 1.3. Mechanism of Action

The effects of cathinone and its derivatives are mediated through the reversal or inhibition of monoamine reuptake transporters. Cathinone preferentially acts as a catecholamine inhibitor and dopamine releaser that has a similar profile to amphetamine [22]. In contrast, mephedrone has demonstrated its action as a nonselective monoamine uptake inhibitor and releaser that is comparable to MDMA [22,23,24,25]. Cathinone and mephedrone activity on serotonin (SERT) and dopamine (DAT) transporters, specifically in the nucleus accumbens [26,27,28,29], may contribute to their psychostimulant-like and reinforcing effects, which resemble those of their respective non-βketo analogues, amphetamine and MDMA [23,30,31]. Methylone has demonstrated lower neuronal activity inhibition potency compared to MDMA, which may be caused by the addition of a ketone group for ethylone [32]. According to their selectivity on monoamine transporters, mephedrone and methylone have DAT/SERT ratios between 0.1 and 10, which are higher ratios than those obtained for MDMA. High selectivity on DAT with >10 DAT/SERT ratios may suggest increased abuse potential, especially given that dopamine has been related to reinforcing effects [33,34]. Although diethylpropion also induces dopamine efflux and slows dopamine reuptake in the nucleus accumbens and striatum [35], its effects are less potent compared to cocaine, bupropion, and ethcathinone [36].

### 1.4. Pharmacological Effects

Khat produces a plethora of effects that can mainly be attributed to cathinone and, to a lesser extent, other components of the plant, such as cathine [5,37]. Principally, khat chewing produces stimulant-like effects that are characterized by feelings of mild euphoria, increased alertness, and enhanced intellectual efficiency. Mild dysphoria, irritability, anorexia, and insomnia commonly appear as after effects [5,6,38].

Regarding mephedrone, it is principally consumed via the oral and intranasal routes, with usual doses ranging from 15 to 300 mg and 5 to 125 mg, respectively [39]. Mephedrone induces a spectrum of sympathomimetic and psychostimulant effects that mainly include euphoria, increased energy, mood enhancement, empathy, sociability, and changes in perception [40,41,42].

Methylone, similar to mephedrone, can be administered via multiples routes, although the oral route is the most common. Recreational users have reported that moderate oral doses (100–200 mg) induce a typical profile of euphorigenic and stimulant effects [43].

Diethylpropion was approved for treatment of obesity by the FDA in 1959 and has been used to achieve modest weight loss [44]. This synthetic cathinone is classified as a Schedule IV controlled substance in the United States and Canada since it has an accepted medical use. Nevertheless, although it has reduced stimulant activity and presumably low abuse potential compared to amphetamines [45], addiction should not be discarded.

To date, there are few studies concerning the human pharmacology and abuse potential of cathinone and its synthetic analogues. The main purpose of this systematic review is to therefore examine and compare the pharmacological effects related to the abuse potential and pharmacokinetic properties of cathinone and synthetic cathinones, such as mephedrone, methylone, and diethylpropion, as evaluated in experimental and prospective observational studies in humans.

## 2. Materials and Methods

This systematic review was conducted in accordance with the PRISMA (Preferred Reporting Items for Systematic Reviews and Meta-Analyses) guidelines. It has been submitted in PROSPERO with the registration ID CRD42021271851.

First, eligible articles were systematically looked for and identified to select potential studies by two of the authors (A.T. and L.P.). Study selection was performed jointly, and in the cases of a discrepancy, the final decision was reached by the consensus of either both authors or with the involvement of a third person (M.F.) after reading the study summary. Data were collected by the authors A.T. and L.P. and were subsequently reviewed by another (M.F.).

The published literature search was conducted with the PubMed database and covered the period up to September 2021. The keywords that were employed included “khat”, “mephedrone”, “methylone”, “diethylpropion”, “cathinone”, “synthetic cathinone”, “abuse potential”, “abuse liability”, “experimental study”, “observational study”, “naturalistic study”, “clinical trial”, “randomized clinical trial”, “pharmacological effect”, “subjective effect”, and “physiological effect”. Only articles whose abstracts met our selected criteria were included. The established inclusion criteria were experimental/observational studies evaluating the pharmacological effects related to the abuse potential of cathinone or its synthetic derivatives mephedrone, methylone, and diethylpropion in humans. Studies were considered eligible irrespective of the route used for substance administration and regardless of whether there was a control condition. Second, studies were excluded when they focused on animal models, were retrospective, and provided results obtained from a survey in which participants were not required to use the substance in order to respond (to be eligible, effects had to be evaluated during/immediately after administration/self-administration). Additionally, studies were excluded if they only provided the pharmacokinetics results without evaluating the pharmacological effects. Publications identified as a commentary, editorial, or review were also excluded.

Bupropion has a low abuse potential compared to amphetamines and other cathinones. The results of epidemiological studies show a very low prevalence of bupropion abuse. Bupropion is not a controlled substance. For these reasons, its inclusion in this systematic review was not contemplated, and studies that focused on this substance alone were excluded.

The following information was obtained from the selected articles: study design, sample size, dose, and route of administration; pharmacokinetic parameters in plasma/blood or oral fluid (if measured), such as maximum concentration (Cmax), time to achieve maximum concentrations (Tmax), and area under the curve (AUC); pharmacological effects related to abuse potential; and assessment tools for measuring those effects.

Additionally, when possible, the correlation between the administered dose and the Cmax of the substance in blood was calculated.

## 3. Results

**Figure 2:**
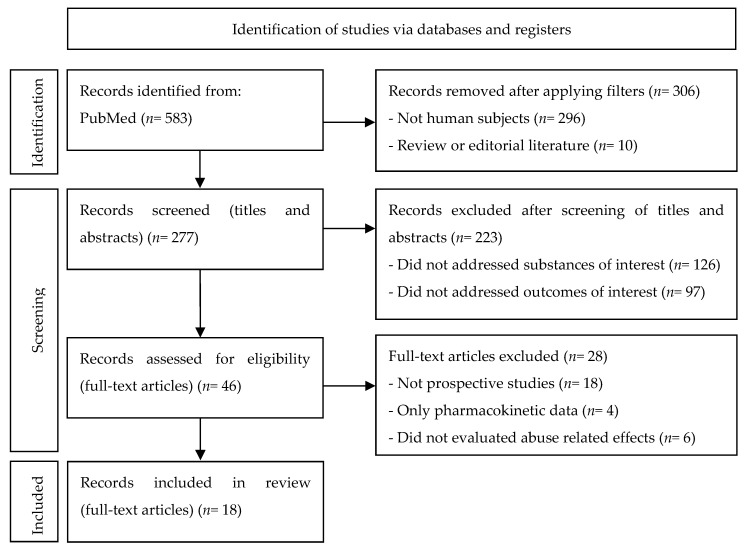
PRISMA flow chart for the study selection and data extraction.

In total, 583 results were obtained from the literature search, and 18 publications were selected after meeting the inclusion criteria (Figure 2). Table 1, Table 2, Table 3 and Table 4 summarize the information retrieved from the selected studies about the human pharmacology and abuse-related effects of cathinone, mephedrone, methylone, and diethylpropion, respectively. All of the included studies provided results about the pharmacological effects related to abuse, and some of them also included the findings regarding pharmacokinetics. In the case of publications by the same authors that contained duplicated information about the study design, sample size, and administered dose, their results about the pharmacokinetics and pharmacological effects were unified in the corresponding table.

From the accessible full-text articles about experimental studies that were screened, four were excluded for only including the pharmacokinetic data regarding the pharmacological effects of cathinone [46], diethylpropion [47], and mephedrone [48,49] without providing results. Another six studies were excluded since they evaluated pharmacological effects that were not related to the abuse potential of cathinone [9,50] or diethylpropion [51,52,53,54].

In the majority of the studies assessing the abuse potential of cathinone, mephedrone, and methylone, the participants were healthy individuals with previous psychostimulant use. Overall, most studies included small sample sizes, and not all of them comprised both genders.

### 3.1. Cathinone

In total, we retrieved five publications (four studies) concerning cathinone administration in humans (Table 1) [4,8,20,31,55]. According to this search, four studies analyzed the pharmacokinetic properties of cathinone, which is the component responsible for the psychoactive properties of khat. In three studies, participants chewed khat leaf preparations for 1 h in doses from 0.5 to1.0 mg of cathinone per kg of body weight, which corresponds to the most popular and traditional route of administration. The mean Cmax of cathinone in the blood after khat-chewing ranged from 58.9 ng/mL to 127 ng/mL when obtained from doses of 0.684 mg/kg and 0.8 mg/kg, respectively, with a Tmax from 1.2 to 2.31 h.

In another study, cathinone was orally administered in the form of gelatin capsules. They contained 0.5 mg cathinone per kg of body weight and were compared to placebo capsules. According to the figure showing the time-course of cathinone concentrations in blood, the maximum value of the time-course was approximately 105 ng/mL, and the Tmax was at 1.2 h post-administration.

Regarding abuse-related effects, cathinone produced an increase in the participants’ blood pressure and heart rate, and the most common subjective effects were euphorigenic ones, increased energy, and motor stimulation.

### 3.2. Mephedrone

A total of seven publications, corresponding to five studies, about the pharmacokinetic and acute effects of mephedrone in humans were retrieved (Table 2) [56,57,58,59,60,61,62]. Out of the six studies, five evaluated mephedrone concentrations in the blood or oral fluid, while the other only collected the pharmacological effects reported by the participants after using unknown doses. Specifically, four studies analyzed the time course of the mephedrone concentrations in the blood, whereas oral fluid was used as an alternative matrix in an observational study. In one study, mephedrone was also administrated in combination with alcohol (0.8 g/kg) to assess their interaction and impact on the subjective, cardiovascular, and hormone effects as well as the pharmacokinetics. Together with the evaluation of abuse-related effects, the mephedrone concentrations in blood and oral fluid were studied after oral administration in four studies (doses from 50 to 200 mg) and after intranasal administration in an observational one (doses from 50 to 100 mg, mean dose 70 mg).

The Cmax following the oral administration of mephedrone seemed to be dose dependent. The lowest oral dose of 50 mg resulted in a Cmax of 37.4 ng/mL, whereas the highest Cmax value of 255.6 ng/mL corresponded to 200 mg. In general, the maximum concentrations of mephedrone reached between 1 and 1.5 h after oral administration.

Studies that evaluated the pharmacological effects of mephedrone found its stimulant effects to be characterized by an increase in the cardiovascular effects and pupillary diameter, euphoria, mild changes in perceptions, and memory impairment.

**Figure 3:**
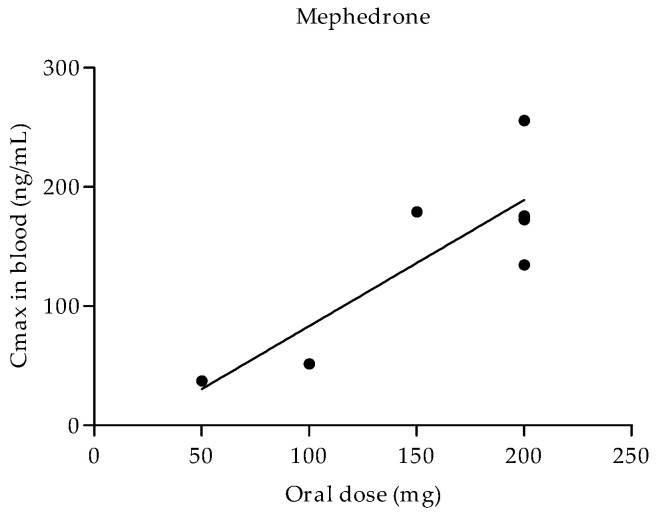
Correlation between oral mephedrone dose and the maximum concentration reached in blood.

In the case of mephedrone, it was possible to calculate a correlation between single orally administered doses of mephedrone and the Cmax achieved in the blood. Oral doses of mephedrone and their respective Cmax in the blood showed a moderate correlation with a Pearson’s *r* of 0.8353 and a coefficient of determination of 0.6977 (*p* value of 0.0193) (Figure 3).

### 3.3. Methylone

Only one study concerning methylone administration in humans was found in the literature (Table 3) [63]. This observational study was carried out in naturalistic settings, where eight participants selected their oral methylone doses from between 100 to 300 mg (mean dose 187.5 mg) according to their experience with psychostimulant use (Poyatos, 2021). A total of six participants self-administered a single oral dose of 75 or 100 mg of MDMA. Concentrations of methylone were analyzed in the oral fluid, obtaining a Cmax of 15,514.0 and a Tmax of 2 h. The self-administration of methylone produced an increase in the systolic blood pressure and heart rate, accompanied by psychostimulant-like effects such as “stimulation”, “high”, and “good effects”.

### 3.4. Diethylpropion

Regarding diethylpropion, five publications (six studies) were retrieved (Table 4) [64,65,66,67,68]. Among the selected studies, diethylpropion was administered to compare it to d-amphetamine [65]; d-amphetamine and lisdexamfetamine [68]; pipradrol and amobarbital [64]; and dexamphetamine, phenmetrazine, and caffeine [64] in four studies.

Two studies assessed participant preferences for diethylpropion compared to placebo or d-amphetamine. This type of research is considered the paradigm of abuse potential assessment.

Diethylpropion produced effects that were qualitatively similar, but less intense, to those of d-amphetamine, with euphoria, increased blood pressure and body temperature, and decreased food intake being observed. Some adverse effects such as anxiety, irritability, dry mouth, and insomnia did, however, appear within the first three months of treatment.

## 4. Discussion

To the best of our knowledge, this is the first systematic review that is aimed at investigating and comparing the abuse-related effects and pharmacokinetic profiles of cathinone and other synthetic derivatives such as mephedrone, methylone, and diethylpropion. Our search was focused on cathinone, which is the main compound of the khat plant, and three synthetic cathinones, one of which was approved for therapeutic purposes and two others that are considered substances of abuse. Information regarding the abuse potential of synthetic cathinones is scarce despite representing the second largest group monitored by the European Union Early Warning System and their relevant impact on public health and security. Synthetic cathinones have been related to several cases of intoxication and fatalities over the years [69]. The variety of these compounds on the market is constantly growing; nevertheless, few components of this group have been researched in humans. Moreover, the abuse potential of synthetic cathinones assessed in various preclinical animal models has only been reviewed recently [70].

The studies included in this systematic review provided data about the subjective effects related to the abuse potential of cathinone and some of its synthetic derivatives. They employed validated methods recognized by the FDA and Health Canada for the assessment of abuse potential [1]. Most of the studies evaluated acute reinforcing effects of substances through ARCI and/or VAS, whereas others incorporated VESSPA, POMS, and additional specific questionnaires. Self-administration studies about drug preference were also included, and they directly evaluated whether the substance had rewarding or reinforcing properties. Drug discrimination questionnaires, such as the pharmacological class identification questionnaire, were used in some studies to assess whether the drug had effects that were similar to other known drugs of abuse.

### 4.1. Cathinone

In the majority of studies involving cathinone, the selected route of administration was chewing khat leaves for 1 h. As it has been previously mentioned, this is, by far, the most popular way of cathinone administration due to its traditional background in several African and West Asian countries. Cathinone that was ingested by khat-chewing demonstrated slower pharmacokinetics and the delayed onset of pharmacological effects compared to when cathinone was contained in capsules. This was mainly due to the delayed release of the alkaloid from the leaf matrix during mastication [4]. During chewing, the buccal mucosa was responsible for most of the absorption of the three alkaloids [20]. Such absorption allows the drug to enter directly into systemic circulation, bypassing the gastrointestinal tract and first-pass metabolism in the liver. A second absorption of the swallowed juice, however, takes place in the stomach or small intestine.

In some studies, cathine and norephedrine, the main metabolites of cathinone as well as the components of the khat plant [71], were analyzed. Although these metabolites are active compounds and may contribute to psychoactive effects, their action is less potent than that of cathinone [37,72].

### 4.2. Mephedrone

According to our search, mephedrone is the most investigated synthetic cathinone in humans. Mephedrone users reported consuming this substance as a substitute for MDMA due to its similar pharmacological effects. The pharmacological profile and pharmacokinetics of mephedrone were evaluated following oral and intranasal administration, both of which are considered the most conventional routes.

In terms of oral administration, mephedrone was employed in comparison with MDMA, which was used as a prototypical psychostimulant reference [57]. Under controlled conditions, an oral dose of 200 mg mephedrone produced noticeable cardiovascular effects and induced stimulant-like and pleasurable effects that were comparable to those produced by MDMA. In comparison to 100 mg of MDMA, the pharmacological effects of mephedrone had an earlier onset and faster disappearance. This event may be related to the brief elimination half-life of mephedrone, which could lead to a compulsive consumption pattern to maintain the desired effects in real life conditions.

Mephedrone administration has been also studied in combination with alcohol, the substance it is most commonly combined with on the nightlife scene. In this study, 200 mg of mephedrone was administered in combination with 0.8 g/kg alcohol or placebo [58,59]. The concomitant administration of mephedrone and alcohol amplified the cardiovascular effects and induced more intense and prolonged feelings of euphoria and well-being associated with mephedrone. In turn, mephedrone reduced the drunkenness and sedation produced by alcohol, although this apparently improved state was not reflected in the psychomotor performance. In general, such improved subjective effects could suggest the greater abuse potential of the simultaneous consumption of mephedrone and alcohol in addition to a heightened risk of serious adverse effects associated with both.

The most popular mephedrone administration route is insufflation, and few studies have analyzed the pharmacokinetics of mephedrone and its metabolites in human plasma and oral fluid after intranasal administration [48,49,62]. As expected, mephedrone concentrations reached its maximum levels in the blood earlier after the intranasal administration of 100 mg (Tmax of 0.88 ± 0.35 h) [49] compared to the oral administration of the same dose (Tmax of 1 h ranging from 1 to 2 h) in laboratory conditions [60]. This rapid absorption produces fast and short-lasting pharmacological effects, which often lead to a pattern of repetitive use in users intending to prolong the desired effects. In an observational study comparing these two routes, oral self-administration produced greater and larger effects on some subjective measures [62]. The authors did, however, comment that the subjective effects after intranasal self-administration could be underestimated due to the first evaluation (at 1 h) being conducted after the expected maximum subjective effects were induced. Regarding the oral fluid concentrations of mephedrone, those were considerably higher after intranasal administration compared to oral administration [62].

### 4.3. Methylone

In a similar manner, methylone, another synthetic cathinone of abuse potential comparable to mephedrone, has been shown to produce the prototypical psychostimulant and empathogenic effects associated with MDMA. These include euphoria, stimulation, perception alteration, energy increase, and sociability, although to a lesser extent. The results suggest that methylone induced a similar profile of effects to its non-β-analogue MDMA and mephedrone [62,63], both of which were also administered in naturalistic conditions but showed lower intensity of effects.

### 4.4. Diethylpropion

In contrast to cathinone, mephedrone, and methylone, which are considered substances of abuse, diethylpropion, due to its relatively lower abuse potential compared to amphetamine, has been commercialized as an appetite suppressant and obesity treatment under the trade names of Tenuate^®^ and Tepanil^®^. Tenuate^®^ has, however, currently been discontinued on the North American market according to the FDA. The efficacy of diethylpropion for the treatment of overweight and obese patients has been previously discussed [44,73,74].

Most studies regarding diethylpropion have focused on its therapeutic effects, for instance, as an anorectic agent. A few other studies have, however, evaluated the abuse potential of this synthetic cathinone i compared to other substances, such as d-amphetamine [65,66] and lisdexamfetamine [68]. One of them aimed to assess participant preferences between diethylpropion and d-amphetamine at different doses and to measure the reinforcing properties of these drugs. Although the results demonstrated that the subjects generally preferred both amphetamine and diethylpropion to placebo, diethylpropion seemed to show weaker reinforcing properties in comparison to amphetamine [66]. In terms of psychotropic effects, diethylpropion produced effects that were qualitatively similar to those of d-amphetamine, with feelings of euphoria and increased blood pressure being observed, although with less intensity [65,68]. Compared to lisdexamfetamine, there were no statistically significant differences in terms of abuse-related liking scores [68].

In a study evaluating diethylpropion, healthy participants received a single oral dose of 75 mg, and its pharmacokinetics were assessed according to the metabolizer profile, distinguishing among slow, intermediate, normal, and fast metabolizers [47]. The means of the main pharmacokinetic parameters obtained from each metabolizer phenotype were significantly different, with an approximately threefold variation between the slowest (Cmax 11.07 ng/mL and AUC0-t 48.84 ng/mL·h) and fastest (Cmax 4.12 ng/mL and AUC0-t 15.30 ng/mL·h) phenotypes.

Overall, in terms of pharmacological effects, cathinone, diethylpropion, mephedrone, and methylone shared a similar stimulant profile to other recreational substances, such as MDMA and amphetamine [57,63,75]. These substances commonly produce an increase in cardiovascular effects and body temperature as well as stimulant and euphorigenic effects. The greater the intensity of the rewarding effects, the higher the abuse potential (cathinone and mephedrone).

The correlation between the oral administration dose and Cmax in blood is the only possible method through which mephedrone could be calculated since the pharmacokinetic data of cathinone, methylone, and diethylpropion were insufficient. Doses of mephedrone that were administered orally through capsules and Cmax in the blood presented a significant moderate to strong correlation. This suggests that the absorption of mephedrone is constant at these evaluated doses, which may prove useful for predicting or extrapolating the Cmax value at any mephedrone dose between the evaluated ranges.

### 4.5. Limitations

This systematic review has some limitations that should be considered. Firstly, only one database, PubMed, was consulted for publication retrieval. Moreover, the search was limited to English. Inclusion was restricted to prospective studies, so the results obtained from retrospective studies, such as surveys about previous consumption, were not accepted. Due to the scarcity of studies about the abuse potential of synthetic cathinones in humans, only those concerning mephedrone, methylone, and diethylpropion were included in this review. The variety of study designs hindered the identification of consistent differences among the compared substances. We included both experimental placebo-controlled trials and observational prospective studies. Whilst it may be a limitation, inclusion was based on the fact that evaluation was performed with similar questionnaires and methods to assess abuse potential.

## 5. Conclusions

In conclusion, despite the large number of existing synthetic cathinones and the constant appearance of other derivatives, research about their abuse potential and pharmacokinetics is still very limited. Mephedrone is the most studied synthetic cathinone used for recreational purposes; little is known, however, about the great majority of other synthetic cathinones. Regardless of their legal status, cathinone and its synthetic derivatives induce a range of desirable and reinforcing effects that may result in some degree of abuse potential. All four substances share a similar psychostimulant profile that is close to the profiles of amphetamines and MDMA. The different intensities of the desirable effects they present may be associated with varying levels of abuse potential. Mephedrone showed a moderate correlation between the administration dose and Cmax in experimental studies administering single oral doses.

Further investigation is needed to shed light on the abuse potential of cathinone and its synthetic derivatives to minimize the impact of their consumption on society and to detect and prevent any medical complications associated with their use and abuse.

## Figures and Tables

**Table 1:** Summary of studies evaluating abuse-related effects related to cathinone administration in humans included in this systematic review.

Reference	Type of Study	Sample Size	Dose	Pharmacokinetics [Mean ± SD, (Range)]	Assessments	Abuse-Related Effects
Nencini et al., 1986 [8]	Experimental, non-controlled, open-label.	14 male volunteers, habitual khat users.	Two khat bundles of 200 g, considered the usual dose for an experienced khat user.	Not reported.	Subjective effects: ARCI, VAS (appetite, vigilance and euphoria/dysphoria). Physiological effects: Supine SBP, DBP and HR.	Subjective effects: 10 subjects experienced euphoria and increased intellectual efficiency and alertness (ARCI MBG, A, and BG). These effects were progressively replaced by mild dysphoria and sedation (ARCI LSD and PCAG). The other four subjects reported minimal amphetamine-like effects although they also experienced dysphoric effects. CATH showed mild anorectic effects. Physiological effects: CATH increased SBP, DBP, and HR in all the subjects.
Brenneisen et al., 1990 [31]	Experimental, placebo- controlled, double-blind, randomized and crossover study.	Six healthy male volunteers.	Orally administered gelatin capsules of 0.5 mg CATH/kg body weight. Placebo	Data deduced from a figure. CATH: Cmax: approx. 105 ng/mL AUC_0–9_: 307 ± 71 ng/mL·h * Tmax: 1.2 ± 0.55 h * T1/2: 4.81 ± 1.05 h * Norephedrine: Cmax: approx. 75 ng/mL AUC_0–9_: 489 ± 228 ng/mL·h * Tmax: 2 h	Subjective effects: ARCI. Physiological effects: SBP, DBP, and HR were monitored.	Subjective effects: CATH induced psychostimulant and euphorigenic effects, reflected by an increase in scores for ARCI stimulation/ euphoria and amphetamine-like effects. Physiological effects: CATH produced an increase in blood pressure and HR.
Widler et al., 1994 [4]	Experimental, placebo-controlled, double blind.	Six healthy males without previous khat chewing experience.	0.8 mg cathinone/kg body weight (54 to 71 g fresh khat leaves) of a standardized preparation of khat leaves chewed for 1 h. CATH: 1.02 ± 0.11 mg Cathine: 0.86 ± 0.06 mg Norephedrine: 0.47 ± 0.05 mg Placebo: alkaloid-free khat.	CATH: Cmax: 127 ± 53 ng/mL AUC_0–9_: 415 ± 207 ng/mL·h Tmax: 2.12 ± 0.5 h Cathine: Cmax: 89 ± 51 ng/mL AUC_0–9_: 466 ± 299 ng/mL·h Tmax: 3.05 ± 1.22 h Norephedrine: Cmax: 110 ± 51 ng/mL Tmax: 3.33 ± 2.23 h AUC_0–9_: 633 ± 337 ng/mL·h	Subjective effects: ARCI, VAS. Physiological effects: SBP, DBP, and HR were continuously monitored.	Subjective effects: CATH increased scores in the amphetamine–effect, stimulation–euphoria, and stimulation–motor ARCI scales. Subjects reported feeling more excited and energetic in VAS. Physiological effects: CATH produced a significant mild and slow increase in SBP and DBP that persisted for 4 h. The increase in HR was not significant.
Toennes et al., 2002; 2003 [20,55]	Experimental, non-controlled, open label.	Four (two M, two F) healthy, without previous khat chewing experience	0.6 g of khat leaves per kg body weight chewed for 1 h: CATH: 1.14 mg/g khat (0.684 mg cathinone/kg body weight) Cathine: 0.83 mg/g khat Norephedrine: 0.44 mg/g khat	Blood concentrations: CATH: Cmax: 58.9 ± 18.8 ng/mL AUC: 245 ± 49 ng·min/L Tmax: 2.31 ± 0.65 h t ½ α: 0.39 ± 0.07 h t ½ β: 1.50 ± 0.81 h Cathine: Cmax: 71.2 ± 13.9 ng/mL AUC: 713 ± 131 ng·min/mL Tmax: 2.62 ± 0.77 h t ½ α: 0.24 ± 0.17 t ½ β: 5.22 ± 3.36 Norephedrine: Cmax: 72.1 ± 12.2 ng/mL AUC: 710 ± 173 ng·min/mL Tmax: 2.84 ± 0.42 h Urine concentrations: CATH: Cmax: 2.5 mg/L Cathine: Cmax: 20 mg/L Norephedrine: Cmax: 30 mg/L	Subjective effects: The mental state of the subjects was assessed using a list of paired terms describing opposite states of emotion (“Befindlichkeitsskala”). Physiological effects: SBP, DBP, HR, PD, and rotator nystagmus. Reaction to visual and acoustic stimuli was tested using the Wiener Determinations test, and the individual attention and concentration performance was tested using Test d2.	Subjective effects: All participants reported the personal feeling of being alert and “energetic”. Physiological effects: Participants experienced an increase in blood pressure that might not be caused by the pharmacological action of the alkaloids. HR, PD, and reaction to light showed no changes. Rotatory nystagmus and an impairment of mental condition were not observed.

* Data published in [4].

**Table 2:** Summary of studies evaluating abuse-related effects related to mephedrone administration in human included in this systematic review.

Reference	Type of Study	Sample Size	Dose	Pharmacokinetics [Mean ± SD, (Range)]	Assessments	Abuse-Related Effects
Freeman et al., 2012 [56]	Observational-naturalistic controlled, open label	20 MEPHE users (14 M, 6 F) and 20 controls drug free (11 M, 9 F)	Not reported. MEPHE users self-administered the drug as they normally would (dose and route of administration).	Not reported.	Subjective effects: VAS, BDI, O-LIFE, and MFUQ. Physiological effects: - Cognitive assessments: Prose recall (rivermead behavioral memory test), spatial N-back, phonological fluency, semantic fluency, trail making test, Wechsler adult reading test.	Subjective effects: MEPHE produced an increase in stimulant effects, particularly “self-confidence”, “buzzing”, and “dizziness”. Physiological effects: - Cognitive assessments: MEPHE impaired concentration and memory and also enhanced psychomotor speed.
Papaseit et al., 2016 [57]	Experimental placebo- controlled double-blind, double dummy, randomized, and crossover trial	12 healthy males who were recreational users of amphetamines MDMA, MEPHE, and cathinones.	200 mg of oral MEPHE 100 mg of oral MDMA Placebo	Cmax: 134.6 ± 63.5 ng/mL AUC_0–12_: 519.5 ± 287.0 ng/mL·h AUC_0–24_: 556.2 ± 320.2 ng/mL·h AUC_0–inf_: 556.2 ± 320.2 ng/mL·h Tmax: 1.25 (0.5–4) h Ke: 0.33 ± 0.07 per h t1/2: 2.15 ± 0.4 h	Subjective effects: VAS, VESSPA, ARCI, and pharmacological class identification questionnaire. Physiological effects: Non-invasive SBP, DBP, HR, and PD. Electrocardiogram was continuously monitored.	Subjective effects: MEPHE induced stimulant-like effects, euphoria, and well-being and induced mild changes in perceptions comparable to those of MDMA. Physiological effects: MEPHE increased SBP, DBP, HR, and PD.
De Sousa et al., 2016 [58]; Papaseit et al., 2020 [59]	Experimental placebo-controlled, double-blind, randomized, crossover phase I clinical trial.	11 healthy males, recreational users of amphetamines, MDMA, MEPHE, or cathinones.	200 mg of oral MEPHE + 0.8 g/kg alcohol 200 mg of oral MEPHE + placebo alcohol Placebo MEPHE + 0.8 g/kg alcohol Placebo MEPHE + placebo alcohol	MEPHE alone Cmax: 172.6 ± 82.9 ng/mL AUC_0–6_: 549.0 ± 315.0 ng/mL·h AUC_0–24_: 778.4 ± 512.9 ng/mL·h Tmax: 1.5 (0.5–2) h Ke: 0.29 ± 0.09 per h t1/2: 2.68 ± 0.92 h MEPHE with alcohol Cmax: 175.7 ± 71.1 ng/mL AUC_0–6_: 516.8 ± 264.6 ng/mL·h AUC_0–24_: 709.8 ± 477.1 ng/mL·h Tmax: 1.5 (0.75–2) h Ke: 0.35 ± 0.14 per h t1/2: 2.32 ± 1.01 h	Subjective effects: VAS, VESSPA, ARCI, and pharmacological class identification questionnaire. Physiological effects: SBP, DBP, and HR were continuously monitored. Oral temperature was measured, and DP and the Maddox wing were recorded. Neurocognitive assessment: SMT, CTT, DAT.	Subjective effects: MEPHE induced stimulant-like effects (euphoria, well-being, feelings of pleasure) and mild changes in perceptions that were more intense and prolonged in combination with alcohol. Physiological effects: MEPHE produced a significant increase in BP, HR, and PD. Cardiovascular effects were increased in combination with alcohol. Neurocognitive assessment: MEPHE improves psychomotor performance, impairs spatial memory, and does not affect divided attention performance.
Olesti et al., 2017; 2019 [60,61]	Experimental double-blind, placebo controlled, randomized, crossover, phase I clinical trial.	Nine healthy males, recreational users of NPS.	50 and 100 mg of oral MEPHE (*n* = 3) 150 and 200 mg of oral MEPHE (*n* = 6)	MEPHE 50 mg Cmax: 37.4 ± 16.4 ng/mL AUC_0–8_: 122.5 ± 59.7 ng/mL·h Tmax: 2 (1–2) h MEPHE 100 mg Cmax: 51.7 ± 20.5 ng/mL AUC_0–8_: 169.4 ± 93.5 ng/mL·h Tmax: 1 (1–2) h MEPHE 150 mg Cmax: 179.0 ± 29.3 ng/mL AUC_0–8_: 588.2 ± 93.4 ng/mL·h Tmax: 1 (1–2) h MEPHE 200 mg Cmax: 255.6 ± 70.0 ng/mL AUC_0–8_: 879.4 ± 194.1 ng/mL·h Tmax: 1 (1–2) h	Subjective effects: VAS. Physiological effects: SBP, DBP, and HR were continuously monitored.	Subjective effects: MEPHE induced subjective effects (VAS high, good effects, stimulated) that showed a positive correlation with drug concentrations in the plasma at each tested MEPHE dose. Physiological effects: MEPHE produced cardiovascular effects that correlated positively with drug concentrations in the plasma at each tested MEPHE dose.
Papaseit et al., 2021 [62]	Observational-naturalistic, non-controlled, open label.	10 (4 F, 6 M) healthy subjects, recreational users.	Self-administration of oral MEPHE (*n* = 5, 100–200 mg; mean 150 mg) Self-administration of intranasal MEPHE (*n* = 5, 50–100 mg, mean 70 mg)	Oral fluid concentrations Oral MEPHE: Cmax: 1571 ± 1367 ng/mL AUC_0–4_: 3684 ± 3443 ng/mL·h Tmax: 2 (1–2) h Intranasal MEPHE: Cmax: 4950 ± 5545 ng/mL AUC_0–4_: 7919 ± 7717 ng/mL·h Tmax: 1 (1–1) h	Subjective effects: VAS, VESSPA, ARCI. Physiological effects: Non-invasive SBP, DBP, HR, and cutaneous temperature.	Subjective effects: MEPHE oral self-administration in comparison to intranasal produced greater and larger effects on some subjective measures. Physiological effects: Both MEPHE self-administrations produced an increase in SBP, DBP, HR.

**Table 3:** Summary of studies evaluating abuse-related effects related to methylone administration in human included in this systematic review.

Reference	Type of Study	Sample Size	Dose	Pharmacokinetics [Mean ± SD (Range)]	Assessments	Abuse-Related Effects
Poyatos et al., 2021 [63]	Observational- naturalistic non-controlled, open label.	14 (4 F, 10 M) healthy subjects, recreational users.	Self-administration of oral METHY (*n* = 8, 100–300 mg; mean 187.5 mg) Self-administration of oral MDMA (*n* = 6, 75–100 mg, mean 87.5 mg)	Oral fluid concentrations: Cmax: 15,514.00 ± 9748.86 ng/mL AUC_0–4_: 40,623.79 ± 20,001.70 ng/mL·h Tmax: 2 (2–2) h	Subjective effects: VAS, VESSPA, ARCI. Physiological effects: Non-invasive SBP, DBP, HR, and cutaneous temperature.	Subjective effects: METHY induced similar psychostimulant and empathogenic effects to MDMA, but they were less intense. Physiological effects: METHY produced an increase in SBP and HR.

**Table 4:** Summary of studies evaluating abuse-related effects related to diethylpropion included in this systematic review.

Reference	Type of Study	Sample Size	Dose	Pharmacokinetics [Mean ± SD, (Range)]	Assessments	Abuse-Related Effects
Jonsson et al., 1969 [64]	Double-blind study in natural environment.	116 subjects of both genders.	25, 50 mg of oral DEP 20 mg of oral pipradrol 100 mg of oral amobarbital Placebo	Not reported.	Subjective effects: Scales comprising 20 variables (e.g., happiness, alertness, relaxation, flight of thoughts), Physiological effects: -	Subjective effects: Relative to placebo, both doses of DEP gave results in the same direction as pipradrol. Compared to placebo, DEP obtained higher scores for “happy”, “alert”, and “flight of thoughts” and lower scores in “relaxed”. Physiological effects: -
Jonsson et al., 1969 [64]	Experimental, triple-blind study in laboratory conditions.	30 young males.	50 mg of oral DEP 10 mg of oral dexamphetamine 50 mg oral phenmetrazine 250 mg caffeine Placebo	Not reported.	Subjective effects: Scales comprising ratings of pleasantness. Physiological effects: -	Subjective effects: DEP produced stimulant-like effects reflected in feelings of “happiness” similar to dexamphetamine, “alertness”, and “pleasantness”. The effects of “pleasantness” came in between phenmetrazine and dexamphetamine. Physiological effects: -
Jasinski et al., 1974 [65]	Experimental, comparative, placebo controlled, double-blind, randomized, crossover.	nine healthy federal prisoners with documented histories of narcotic abuse. All admitted prior abuse of amphetamine-like agents.	150, 300, and 600 mg of subcutaneous DEP 100, 200, and 400 mg of oral DEP 7.5, 15, and 30 mg of subcutaneous d-amphetamine 10, 20, and 40 mg of oral d-amphetamine Placebo condition	Not reported.	Subjective effects: Drug identifications and “liking scores” from the subject’s and observer’s single-dose opiate questionnaires and ARCI (BG, MBG, A). Physiological effects: SBP, DBP, HR, rectal temperature, and PD.	Subjective effects: DEP produced effects that were qualitatively similar to those of d-amphetamine including euphoria. Orally DEP was 1/6 to 1/11 as potent as d-amphetamine, while subcutaneously, DEP was 1/10 to 1/20 as potent as d-amphetamine. Physiological effects: DEP increased blood pressure and body temperature and decreased caloric intake and sleep.
Johanson et al., 1978 [66]	Drug preference, placebo controlled.	10 (7 M, 3 F) healthy volunteers.	Subjects participated in three to six different choice experiments self-administering oral capsules of: 25, 50 mg of DEP 5, 10 mg of d-amphetamine Placebo	Not reported.	Subjective effects: POMS, choice procedure. Physiological effects: -	Subjective effects: In comparisons between DEP and d-amphetamine, d-amphetamine was generally preferred. However, as the dose of DEP increased, preference for d-amphetamine decreased. Physiological effects: -
Bigelow et al., 1984 [67]	Drug preference, placebo controlled.	Not reported. Overweight patients.	75 mg of DEP Placebo	Not reported.	Subjective effects: Choice procedure. Physiological effects: -	Subjective effects: DEP, with a similar profile to amphetamine, maintained drug preference well above placebo levels (approx. 75%). Physiological effects: -
Jasinski et al., 2009 [68]	Experimental, placebo controlled, double-blind, randomized, six-period crossover study.	38 (32 M, 6 F) volunteers with a history of stimulant abuse. Only 36 completed the study.	200 mg of oral DEP 50, 100, and 150 mg of oral LDX 40 mg of oral d-amphetamine Placebo	Not reported.	Subjective effects: DRQS, ARCI, SVAQ. Physiological effects: SBP, DBP, HR.	Subjective effects: In terms of abuse potential, 200 mg of DEP was appraised as having a comparable street value as 100 and 150 mg of LDX. Physiological effects: All treatments produced cardiovascular effects. Increases in SBP and DBP were significantly lower for 50 mg LDX and DEP than for d-amphetamine.

Abbreviations: Cmax, maximum concentration after administration; Tmax, time needed to reach maximum concentrations; AUC, area under the curve; T1/2, elimination half-life; Ke, elimination rate constant; SD, standard deviations; F, female; M, male; CATH, cathinone; DEP, diethylpropion; LDX, lisdexamfetamine dimesylate; MEPHE, mephedrone; MDMA, 3,4-methylenedioxymethamphetamine; METHY, methylone; BP, blood pressure; SBP, systolic blood pressure; DBP, diastolic blood pressure; HR, heart rate; PD, pupillary diameter; ARCI, Addiction Research Center Inventory; PCAG, pentobarbital–chlorpromazine–alcohol group (sedation); MBG, morphine–benzedrine group (euphoria); BG, benzedrine group (intellectual efficiency); A, amphetamine group (increased energy); VAS, visual analogue scales; VESSPA, Evaluation of Subjective Effects of Substances with Abuse Potential questionnaire; POMS, Profile of Mood States; DRQS, Liking Scale of the Drug Rating Questionnaire-Subject; SVAQ, Street Value Assessment Questionnaire; BDI, Beck depression inventory, O-LIFE, Oxford–Liverpool inventory of feelings and experiences, MFUQ, Mephedrone and future use questionnaire; SMT, Spatial Memory Test; CTT, Critical Tracking Test; DAT, Divided Attention Test.

## Data Availability

Not applicable.
